# On the Superstitions Connected with the History and Practice of Medicine and Surgery

**Published:** 1844-10

**Authors:** 


ART. IV.—*On Superstitions connected with the History and Practice of Medicine and Surgery.* By THOMAS JOSEPH PETTIGREW, F.R.S., F.S.A., Doctor of Philosophy of the University of Göttingen, Surgeon to H. R. H. the Duchess of Kent, to the Asylum for Female Orphans, &c., &c., &c. Philadelphia: Ed. Barrington & Geo. D. Haswell. 1844. 12mo. pp. 213.

This is an entertaining volume, full of instruction for all classes of readers. The bill of fare is suited to the tastes of epicures, but contains articles which, to use the expressive language of Sancho Panza, are both 'toothsome and wholesome' for every variety of constitution. While, therefore, it seems particularly recommended to professional palates, it is much to be desired that it should not be despised by that very large body of readers who give employment to the profession. They would see in it something so nearly akin to some of the popular systems of the present day, that they might, by possibility, have their eyes opened to an important truth. Exploded superstitions, it has been said, never revive, and in the very identical form it may be that they never do. But then it is merely a change of name or shape, without a change of substance—a species of metempsychosis. One superstition 'shuffles off its mortal coil' only to reappear in a more attractive guise, and after a season itself to grow old and perish, to be followed by others in endless succession:—

"So sinks the day star in his ocean bed,
And yet anon repairs his drooping head,
And tricks his beams, and with new spangled ore
Flames in the forehead of the morning sky."

Alchymy and astrology are in their graves—'after life's fitful fever, they sleep well.' But the race is immortal, and *homœopathy* and *hydropathy* flourish in their stead. The butterfly vanishes, but a worm crawls out to take its place. The folly used to be called 'superstition;' in our day it is dubbed 'humbug.' The history of the superstitions which mingled in early medicine is written in the pleasing little work before us: some future historian will relate to wondering posterity how we were led captive by our humbugs.

"We think our fathers fools, so wise we grow;
Our wiser sons, no doubt, will think us so."

Professor Pettigrew divides his work into ten chapters, devoting one to each of the following subjects: *Alchymy*, *Astrology*, *Early Medicine and Surgery*, *Talismans*, *Amulets*, *Charms*, *the Influence of the Mind upon the Body*, *Royal gift of healing*, *Valentine Greatrake's cures*, *Sympathetic cures*. From almost any one of these, it would be an easy matter to quote as many amusing passages as would fill all the space we can devote to the volume. But that which interested us most was the chapter on the *Royal gift of healing*, and in our selection of extracts we shall confine ourselves chiefly to it. We shall see that the testimony in favor of that marvellous gift, was quite equal to anything that has been produced in favor of mesmerism, and we shall also find that some of the first medical men of former times were believers in the reality of the gift. For the first, hear the account of John Browne, one of the surgeons in ordinary to Charless II, and surgeon to his majesty's hospital, as given by our author:

"The author (Browne,) traces the gift of healing from our Saviour to the Apostles, and thence by a continual line of Christian kings and governors, holy men, commencing with Edward the Confessor, whom he regards as the first curer of struma by contact or imposition of hands. John Browne is a great stickler for the limitation of the power of healing by the royal touch to the kings of England; and although he cannot deny to the French king the possession of this faculty, he contends that it has been derived from the English by "sprig of right." With regard to the manner in which the healing was performed, he tells us, that the surgeon having discovered the disease by examination, he grants a certificate to that effect; and tickets being delivered out to the afflicted, they are then presented to his majesty on the surgeon's knee, and he thus delivers every sick person to the king's sacred hand to be touched. The clerk of his majesty's closet first presents a bit of gold to the king, which he receives from the keeper of the closset, upon whose arm the gold medals ready strung are hanging, whilst the chaplain reads the ceremonies and prayers appointed for the purpose, in all which (it is said) 'is shown the great charity, piety, clemency, and humility of our dread sovereign; the admirable effects and wonderful events of his royal cure throughout all nations, where not only English, Dutch, Scotch, and Irish have reaped ease and cure, but French, Germans, and all countreyes whatsoever, far and near, have abundantly seen and received the same; and none ever, hitherto, I am certain, mist thereof, unless their little faith and incredulity starved their merits, or they received his gracious handfor curing another disease, which was not really evermore allowed to be cured by him; and as bright evidences hereof, I have presumed to offer that some have immediately upon the very touch been cured; others not so easily quitted from their swellings till the favor of a second repetition thereof. Some also, losing their gold, their diseases have seized them afresh, and no sooner have these obtained a second touch, and new gold, but their diseases have been seen to vanish, as being afraid of his majesty's presence; wherein also have been cured many without gold; and this may contradict such who must needs have the king give them gold as well as his touch, supposing one invalid without the gift of both. Others seem also as ready for a second change of gold as a second touch, whereas, their first being newly strung upon white riband, may work as well (by their favour.) The tying the Almighty to set times and particular days is also another great fault of those who can by no means be brought to believe but at Good Friday and the like particular seasons this healing faculty is of more vigour and efficacy than at any other time, although performed by the same hand. As to the giving of gold, this only shows his majesties royal well-wishes towards the recovery of those who come thus to be heald. This gold being hereat given as a token of his sacred favour, and pledge of his best desires for them."'

Our author continues the narrative thus—

"The numbers subjected to the royal touch in the reign of Charles II are almost incredible. From a register kept at Whitehall the king appears to have had more patients than all the physicians of his realm. Some were, doubtless, really diseased; but many came for the love of gold, and others from curiosity. The gold was the powerful incentive with many, and instances of attempts to obtain a second piece are narrated. Touching pieces, as they were called, were to be found in the goldsmith's shops, and means were rendered necessary to prevent imposture. The surgeons were required to give certificates to the applicants for relief. According to the Parliamentary Journal for July 2–9, 1660, "the kingdom having been for a long time troubled with the evil, by reason of his majesty's absence, great numbers have lately flocked for cure. His sacred majesty, on Monday last, touched 250 in the banquetting house; among whom, when his majesty was delivering the gold, one shuffled himself in, saying, 'this man hath not yet been touched.' His majesty hath, for the future, appointed every Friday for the cure, at which 200, and no more, are to be presented to him, who are first to repair to Mr. Knight, the king's surgeon, being at the Cross Guns in Russell Street, Covent Garden, over against the Rose Tavern, for their tickets. That none might lose their labour, he thought fit to make it known, that he will be at his home every Wednesday and Thursday, from two to six o'clock, to attend that service; and if any persons of quality shall send to him, he will wait upon them at their lodgings, upon notice given to him.

"The eagerness to obtain certificates from the surgeon was so great, that in Evelyn's 'Diary,' for March 28th, 1684, it is said 'There was so great a concourse of people with their children to be touch'd for the evil, that six or seven were crush'd to death by pressing at the chirurgeon's door for tickets.' (v. i., p. 571.) The number of cases after the restoration appears to have increased greatly, as many as six hundred at a time being touched, and the days appointed being sometimes thrice a week. Some were immediately relieved, others gradually so, and few are reported as not benefitted by the practice. The operation was usually performed at Whitehall upon Sundays, and the success attending it may be judged of by the following curious avowal of the king's surgeon: 'When I consider his majesties gracious touch, I find myself readily *nonplust*, and shall ever affirm, that all chirurgeons whatsoever must truckle to the same, and come short of his marvellous and miraculous method of healing; and for further manifestation hereof, I do humbly presume to assert, that more souls have been healed by his majesties sacred hand in one year, than have ever been cured by all the physicians and chirurgeons of his three kingdoms ever since his happy restoration. Whereas, should an usurper or tyrant surreptitiously, by pride and bloody massacre, forcibly enter his royal throne and touch at the same experiment, you'll never see such happy success; as tryed by the late usurper *Cromwell* in the late rebellious times; influences flow from thence, he having no more right to the healing power than he had to the royal jurisdiction; his tryal rather checquering and darkening the bright rays hereof and so bringing it into obscurity, than affording it any appearance of light.'"

The name of Wiseman is familiar to the student of English surgery. In what relation he stood to this superstition we learn from the following paragraph:

"Serjeant-Surgeon Wiseman, one of the best early English writers upon surgery, bears testimony to the efficacy of the king's touch, and he devotes an entire book to the subject. He strongly contends for the priority of the English kings over those of France in this matter, and he alludes to the controversy of the time of Edward the Confessor, in which it was disputed whether the cure of the evil were a peculiar reward of the king's holiness, or rather a hereditary faculty attending the English crown. Harpsfield, a renowned divine of the Roman persuasion, describes, in his 'Eclesiastical History of England,' a miracle wrought by the Confessor, and says, 'which admirable faculty of curing the struma, he is justly believed to have transmitted to his posterity, the kings of England, and to have continued it amongst them to those times in which he wrote.'* Wiseman says, 'I myself have been a frequent eye-witness of many hundreds of cures performed by his majesties' touch alone, without any assistance of chirurgery; and those, many of them, such as had tyred out the en-

* "Quam Strumosos sanandi admirabilem dotem in posteros suos Anglorum Reges, ad nostra usque tempora transfudisse et perpetuasse, merito creditur." (Hist. Anglicana Ecclesiastica, Duaci, 1622, fol. p. 219.

deavours of able chirurgeons before they came thither. It were endless to recite what I myself have seen and what I have received acknowledgments of by letter, not only from the several parts of this nation, but also from Ireland, Scotland, Jersey, and Guernsey.' And he adds, 'I must needs profess that what I write will do little more than show the weakness of our ability when compared with his majesty's, who cureth more in any one year than all the chirurgeons of London have done in an age.' He points out the cases to which tickets were given for the royal touch. They were to such as have tumours about the neck, and even those accompanied with thick chapped upper lips and eyes affected with a lippitudo."

The healing power does not seem to have been confined to the royal digits of majesty, any more than to particular days. To quote again from Professor Pettigrew—

"Not only were these marvellous healings wrought by the king through the instrumentality of his touch and the present of the angel gold, but it also appears that the blood of the sacred martyr, Charles I, was equally potent in restoring the sick to health. Wiseman refers to the miracles performed by the blood of his majesty shed upon his decollation by the regicides. Drops of blood were gathered on chips and in handkerchiefs by the pious devotees, who could not but think so great a suffering in so honourable and pious a cause, would be attended by an extraordinary assistance of God and some more than ordinary miracles; nor did their faith, says he, deceive them in this point, there being so many hundreds that found the benefit of it. John Browne also relates several instances of this kind, vouched for by persons in high stations of life, some of whom are at this day known to us by their writings and talents."

The history of Mr. Valentine Greatrake is edifying, and as it is brief we copy it entire. Says our author:

"About the middle of the seventeenth century, in the reign of Charles II, Mr. Valentine Greatrake, a gentleman, a member of the Church of England, of great honesty and exemplary sobriety, who is said always to have refused any gratuity for his performances, excited great notoriety, and became, by superstitious feelings, the most celebrated quack of the day. He was a native of Affane, in the county of Waterford, in

Ireland, born of a good family, and had received a good education. In 1649, he entered into the service of the English Parliament, and remained in the army until 1656, when upon the disbandonment of great part of the English, he returned to his native country, and there was engaged in several duties of importance, being registrar for transplantation, a justice of the peace, clerk of the peace for the county of Cork, &c. He lost those places at the time of the Restoration, and it was then, as he describes it, he 'felt an impulse that the gift of curing the king's evil was bestowed upon him.' His impulse was not long confined to the attempted cure of this solitary disease, for, converts rapidly succeeding, he undertook the cure of ague, epilepsy, convulsions, palsy, deafness, and various other complaints all peculiarly under the influence of the nervous system, and all of which were therefore likely to be benefitted by the power exercised over the imagination. He published an account of his cures first in 1666, in the shape of a letter addressed to the Hon. Robert Boyle, accompanied by testimonials from Bishop Wilkins, Bishop Patrick, Dr. Cudworth, Dr. Whichcot, and others of distinction and intelligence. This book is rare, the greater part of the impression having been destroyed in the great fire of London, and it was not reprinted until 1723, by some one who, believing the cures to have been effected by miraculous and supernatural operation, thought it his duty to revive the subject. Those who may be desirous of examining into the cases, may consult the work already referred to, and 'Stubbe's Miraculous Conformist, or an account of severall Marvailous Cures performed by the Stroaking of the Hands of Mr. Valentine Greatarick:'" Oxford, 1666, 4to.

"John Leverett, a gardener, succeeded to the 'manual exercise,' and declared that, after touching thirty or forty a day, he felt so much goodness go out of him that he was as fatigued as if he had been digging eight roods of ground. This is precisely what is now being affirmed by the animal magnetiser."

With the accession to the house of Brunswick the gift of healing by the royal touch seems to have been lost, and we find it now, no longer a regal monopoly, in the democratic hands of the mesmerisers. But it is a curious question, how the touch did work the cures? By imagination, it will be said, which, according to Lord Bacon, 'is next akin to miracle—a working faith.' But it will be objected to this solution of the difficulty that infants were as frequently cured as others. Dr. Heylen, as quoted by our author, declares that he has seen some children 'brought before the king by the hanging sleeves, some hanging at the mother's breasts, and others in the arms of their nurses, all touched, and cured without the help of a serviceable imagination.' We confess we have heard of nothing equal to this, unless it be in the astonishing powers of the metallic tractors, or the magical wand of old Mesmer.

It is not so difficult to explain the *sympathetic* cures. A person being wounded, the surgeon applied the salve, not to the injured part, but to the weapon inflicting the wound, the parts being bound up and protected from the air. How much better it was to apply the unguents thus, than to the wound itself, we at once understand by looking into the composition of some of the weapons-salves of antiquity, among the ingredients of which were human fat and blood, mummy, moss of dead man's skull, bull's blood and fat, and other equally disgusting articles. While these ointments were wrapped about the sword, the *vis medicatrix naturæ* went on unostentatiously to heal the wound by *the first intention.* It was only in wounds of the simplest sort—wounds inflicted by cutting instruments, that this cure was found effectual. It did not succeed in gun-shot wounds, where the process of restoration is different. Our author shows that to this superstition we are indebted for one of the most useful improvements in surgery; and with his remarks on that subject, we take leave of his book, from which we have derived not only entertainment but instruction. He remarks:

"It is not all surprising that cures of the description alluded to should soon be looked upon as the result of magic, incantations, and other supernatural means; and that the professors of the sympathetic art, therefore should have been anxious to account for the effects by natural causes. Such appears to have been Sir Kenelm Digby's chief aim before the doctors of Montpellier, and similar reasonings upon the subject may be found in the writings of the supporters of the system already mentioned, who advocated the plan of treatment and vouched for its efficacy. In this search for natural means to account for the phenomena obtained, the obvious one was overlooked; and the history I have given would be uninteresting but for the valuable practical lesson which these experiments have afforded. We owe to this folly the introduction of one of the first principles of surgery—one which in this country has done more to advance the science than any other beside—one which has saved a vast amount of human suffering and preserved innumerable lives. The history of the doctrine of healing wounds by the power of sympathy is the history of adhesion—the history of union by the first intention—a history which until the time of John Hunter was never fairly developed or distinctly comprehended." Y.

